# Comparative evaluation of cytokine and acute-phase protein concentrations in sera of dairy cows with subclinical and clinical ketosis as a different view of the causes of the disease

**DOI:** 10.14202/vetworld.2021.1572-1578

**Published:** 2021-06-18

**Authors:** Piotr Brodzki, Jan Marczuk, Urszula Lisiecka, Marek Szczubiał, Adam Brodzki, Hubert Gorzkoś, Katarzyna Kulpa

**Affiliations:** 1 Department and Clinic of Animal Reproduction, Faculty of Veterinary Medicine, University of Life Science in Lublin, Lublin, Poland; 2 Department and Clinic of Animal Internal Medicine, Faculty of Veterinary Medicine, University of Life Science in Lublin, Lublin, Poland; 3 Department of Epizootiology and Clinic of Infectious Diseases, Faculty of Veterinary Medicine, University of Life Sciences in Lublin, Lublin, Poland; 4 Department and Clinic of Animal Surgery, Faculty of Veterinary Medicine, University of Life Sciences in Lublin, Lublin, Poland; 5 Private Veterinary Practitioner, Gorvet, Sztutowo, Poland

**Keywords:** acute-phase proteins, cytokine, dairy cows, ketosis

## Abstract

**Background and Aim:**

Ketosis is a common disease occurring during the first stage of lactation in highly productive dairy cows. The aim of the present study was the comparative assessment of selected pro-inflammatory cytokines (including tumor necrosis factor-α [TNF-α] and interleukin 6 [IL-6]), anti-inflammatory cytokines (including IL-10), and acute-phase proteins (APPs) (including haptoglobin [Hp] and serum amyloid A [SAA]), in the sera of cows with subclinical ketosis (SCK), in those with clinical ketosis (CK), and in healthy cows.

**Materials and Methods:**

Thirty dairy cows of Holstein-Friesian breed were investigated. The cows were divided into three groups depending on the serum β-hydroxybutyric acid (βHBA) level. The control, SCK, and CK groups included healthy cows, cows with SCK, and cows with CK, respectively. BHBA concentration in blood serum was determined using colorimetric method. The blood serum was used for proper tests. Cytokine (TNF-α, IL-6, and IL-10) and APPs (SAA and Hp) concentrations in the investigated samples were determined by enzyme-linked immunosorbent assay method.

**Results:**

The SCK group had significantly higher TNF-α, IL-6; IL-10, and SAA values than had the CK group (p<0.01). The SCK group had a lower Hp concentration than had the CK group (p<0.05).

**Conclusion:**

This study showed that the inflammation intensity is higher in the initial phase of the disease and decreases during the advancement, probably due to active anti-inflammatory mechanisms (an increase of IL-10 concentration), which protect animal organism from self-destruction. On the basis of our study, it can be assumed that ketosis development in dairy cows was preceded by the systemic inflammation that may influence the progress of this disease.

## Introduction

Ketosis is a common disease occurring during the first stage of lactation in highly productive dairy cows. Originally, ketosis course is mild without visible clinical symptoms, also known as subclinical ketosis (SCK), which is demonstrated only by an increased concentration of β-hydroxybutyric acid (BHBA) in the blood serum and the presence of ketone bodies in urine. This form of ketosis is typically observed in approximately 40% of cows in a herd; however, in extreme cases, it can involve even 80% of cows [1,2]. The clinical form of ketosis eventually develops without proper intervention, which is characterized by an increased BHBA concentration in the blood, presence of ketone bodies in the urine and milk, and other symptoms, such as poor appetite, rapid weight loss, presence of dry stool, and decline in milk production [3]. Clinical ketosis (CK) may affect approximately 2-15% of cows in a herd in the 1^st^ month of lactation [3]. The disease is a cause of huge economic losses, which arise from the increased susceptibility for infectious diseases, metabolic diseases, and reproductive disorders. In cows with ketosis, loss of appetite, reduced milk yield, and negative changes in milk composition occur, eliminating its utility [4-6]. The basic cause of ketosis is negative energy balance (NEB) that results in the lipolysis of fat reserves with concurrent increased production of ketone bodies. The pathogenesis of the disease includes several different related metabolic processes, such as glycolysis, lipolysis, proteolysis, gluconeogenesis, and amino and fatty acid metabolism. At the start of lactation, the demand for glucose increases in cows because glucose is necessary for the synthesis of milk components. The cow strives to compensate energy deficits and starts to use body fat. The quick lipid mobilization results in an increased concentration of non-esterified fatty acids (NEFAs), wherein after translocation to the liver, is converted to energy (in the form of adenosine triphosphate) that is used by animals for all life processes. However, when glucose deficit occurs, which is utilized in the udder, NEFA does not entirely combust in the Krebs cycle. The excess of NEFA is used for the synthesis of ketone bodies (acetone, acetoacetic acid, and BHBA) and triglycerides that are accumulated in the liver. Increasing milk yield exacerbates energy deficit and adipose tissue disintegration; the concentration of ketone bodies constantly increases inevitably leading to ketosis [7]. During energy deficit, collateral to lipolysis, proteolysis occurs, and the muscle tissue is utilized as a source of amino acids taking an active part in gluconeogenesis [8-11]. The authors of several articles unequivocally point that amino acids are used in the pathogenesis of ketosis in cows [12,13]. Some research revealed a close relationship between ketosis and increased susceptibility for infectious diseases, such as mastitis and urethritis [14,15]. This may suggest a suppressed immunity in cows during ketosis. Pro-inflammatory cytokines and acute-phase proteins (APPs) are generally used as inflammation biomarkers [16-19]. On the basis of the assessment of these markers, it has been demonstrated that a correlation between inflammation and metabolic diseases exists. Moreover, the markers of inflammatory and immune response may play a significant prognostic function as the biomarkers of cows’ health [20,21]. Furthermore, there are reports that metabolic diseases, including ketosis, are preceded by systemic inflammation [2]. In this study, cows with ketosis had increased concentrations of interleukin 6 (IL-6), tumor necrosis factor-α (TNF-α), and haptoglobin (Hp) not only during the diagnosis of the disease but also before parturition, which, according to the authors, suggests the presence of inflammation already during the drying period and early lactation. Other authors even suggest that ketosis development during the perinatal period is preceded by systemic inflammation during the drying period [22]. However, whether inflammation contributes to ketosis in cows remains uncertain. In addition, the pathomechanisms of hyperketonemia in cows with ketosis are unknown [2].

On the basis of the above-mentioned literature data, it was hypothesized that selected parameters of immune system activity in cows may increase with the advancement of the disease. However, if the inflammation develops before and affects ketosis formation, the level of immune parameters will be higher in cows in the first stage of the disease.

The aim of the present study was the comparative assessment of selected pro-inflammatory cytokines (including TNF-α and IL-6), anti-inflammatory cytokines (including IL-10), and APPs (including haptoglobin [Hp] and serum amyloid A [SAA]), in the sera of cows with SCK, in those with CK, and in healthy cows.

## Materials and Methods

### Ethical approval

The study was approved by the Ethics Committee at the University of Life Sciences in Lublin (No. 41/2014).

### Study period and location

The study was conducted from March to June 2014, during the period of the highest number of calvings in the herd, on a private farm located 30 km from Lublin.

### Animals

The research included 30 Holstein-Friesian dairy cows. All the cows included in the study were 10-20-day postpartum. The animals came from a farm of 84 cows, which specialized in milk production. The cows were kept in a free range system, and feeding was based on total mixed ration system. Complete fodder system constituted a proper feed ration, adjusted to the physiological period of the cows. However, the appearance of ketosis may indicate an inadequate feed preparation or insufficient feed consumption by the cows. The feed ration composition in the experimental farm was balanced for lactating cows with average milk production of 24 kg. Moreover, each cow, whose milk yield exceeded 24 kg, additionally received 1 kg of concentrated feed for every 2 kg of additional milk produced. The following were the nutritional dose: Maize silage, haylage, hay, straw, a mixture of cereals, spent grains (Brewers’ grains), protein, and vitamin and mineral supplements. The mixture was administered to the cows once a day after morning milking. Mechanical milking was performed twice a day in a dedicated and properly equipped milking hall. The annual milk yield, determined in the 305-day lactation period, was 8,571-8,670 kg, with fat content of 4.51-4.14%, protein content of 3.14-3.42%, and urea concentration that increased from 141 to 162 mg/L. Herds were free from infectious diseases. Reproductive system control in the herd was regularly conducted at monthly intervals by rectal examination combined with ultrasonography. The cows with no complications during parturition and no signs of inflammation underwent a synchronization protocol of estrus and ovulation (Presynch-Ovsynch protocol) and artificial insemination (AI) with frozen semen. The cows with uterine inflammation were properly treated and subsequently subjected to the synchronization protocol of estrus and ovulation and AI. The cows with ovarian cycle disturbances were individually treated according to the diagnosed cause. Pregnancy check was routinely performed at approximately 30-40 days after AI by rectal examination combined with ultrasonography. The expected date of parturition was determined by adding 280 days from the day of AI, and it was also supported by the pregnancy diagnosis. Screening tests of adult cattle did not reveal the presence of gastrointestinal and pulmonary parasites. Moreover, the testing of feed samples from the total mixed ration system did not show any mycotoxin contents or residues of plant protection products. Initially, all calving cows, up to the 30^th^ day after calving, were subjected to clinical examination and urine test for ketone bodies using the Testoket field test (Biowet, Pulawy, Poland). Among the tested cows, those with a confirmed increased level of ketone bodies in the urine were selected for further treatment. Subsequently, serum BHBA concentrations were determined in all selected cows.

The cows were then divided into appropriate groups on the basis of the BHBA concentrations. The first group consisted of 10 cows with elevated BHBA concentrations (1.6-2.05 mmol/L), without clinical symptoms, and with SCK (SCK group). Moreover, the second group was composed of 10 cows with high BHBA concentrations (3.22-5.6 mmol/L) and with the following symptoms: Decreased or no appetite, weight loss, dry feces, and decreased milk yield (CK group). The control group consisted of 10 healthy cows in the same lactation period, in which ketosis and other diseases were not diagnosed. With respect to the literature data, sick cows (SCK) were considered to be those with serum BHBA concentrations of 1.2-2.9 mmol/L, whereas if the serum BHBA concentration was >3 mmol/L, the cows were considered to have CK. The cows with BHBA concentrations <1.0 mmol/L were classified as healthy. The cows in which the BHBA concentration was 1.0-1.2 mmol/L were considered to be at risk of postpartum-related diseases and thus excluded from our studies [6,23]. No other comorbidities were found in the selected cows. Animals with secondary ketosis (e.g., due to a displaced abomasum) were not included in the study. The blood serum was the material for the actual research. The blood was extracted only once, which was after selecting the cows for the appropriate groups. Similar examinations were performed in all cows selected for the study, including the following selected immunological indicators: Cytokines (IL-6, IL-10, and TNF-α) and APPs (SAA and Hp).

### Blood sampling

Blood samples (9 mL) were extracted from the external jugular vein into Vacutest clot activator tubes, (Vacutest Kima SRL, Arzergrande [PD], Italy). The blood samples were then centrifuged at 2500×g for 10 min at 4°C, and the serum was harvested and transferred to 2 mL microcentrifuge tubes and stored at −80°C until analysis.

### BHBA measurements in the blood serum

BHBA concentrations in the blood serum were determined using the colorimetric method with a reagents kit Ranbut (Randox Laboratories, Crumlin, Antrim, United Kingdom). Intra- and inter-assay coefficients of variation (CV) were 2.8% and 4.7%, respectively. The absorbance readings and subsequent calculations of final concentrations were performed on an automatic microplate reader (Asys Expert Plus, Biochrom Ltd., Cambridge, England) at 450 and 630 nm, respectively.

### Cytokine measurements in the blood serum

The concentrations of TNF-α, IL-6, and IL-10 in the blood serum were determined using commercially available kits, such as bovine enzyme-linked immunosorbent assay kits for TNF-α, IL-6, and IL-10 (USCN Life Science Inc., Houston, USA). The inter- and intra-assay CVs for all examined cytokines were <12% and <10%, respectively. All procedures were performed according to the guidelines provided by the manufacturers and methods available in the literature [24]. Absorbance readings were performed on an automatic microplate reader (Asys Expert Plus, Biochrom Ltd., Cambridge, England) at 450 nm.

### APP measurements in the blood serum

The measurements of SAA concentrations in the blood serum were performed using a commercial enzyme-linked immunosorbent assay kit (Tridelta Development Ltd., Maynooth, Kildare, Ireland). The inter- and intra-assay CVs for SAA analysis were <12.1% and <7.5%, respectively. The determination of Hp in the blood serum was performed using a commercial colorimetric assay kit (Tridelta Development Ltd., Kildare, Ireland). The inter- and intra-assay CVs for Hp analysis were <5.7% and <6.3%, respectively. Procedures were performed according to the manufacturers’ instructions and literature methods [18,25]. Absorbance readings and subsequent calculations of final concentrations were performed on an automatic microplate reader (Asys Expert Plus, Biochrom Ltd., Cambridge, England) at 450 nm and 630 nm for Hp, and 630 nm as a reference for SAA. Lyophilized bovine acute-phase serum was used as a standard, and calibration was performed according to the European Union concerted action on standardization of animal APPs (No. QLK5-CT-1999-0153).

### Statistical analysis

All values were presented as means±standard error of the mean. Statistical analysis was performed using the Statistica software version 10.0 (StatSoft, Poland). Data were found to be normally distributed, as demonstrated by the Kolmogorov–Smirnov test and Lilliefors correction. The obtained values were compared between cows with SCK, cows with CK, and healthy cows, using non-paired Bonferroni *post hoc* multiple comparison test. p<0.05 was considered statistically significant.

## Results

**Figure-1:**
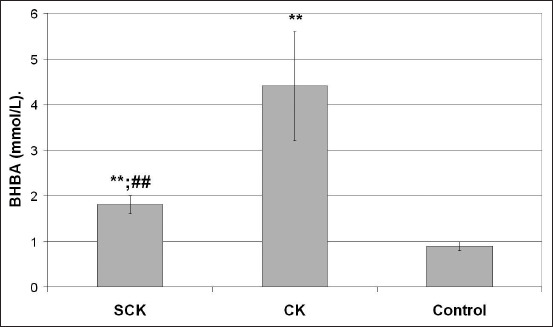
Levels of β-hydroxybutyrate in blood serum of cows with subclinical ketosis (SCK); clinical ketosis (CK); and control (healthy cows). * – p<0.05; ** – p<0.01 – statistical differences between cows with SCK and CK versus control. # – p<0.05; ## – p<0.01 – statistical differences between cows with SCK versus cows with CK.

**Figure-2:**
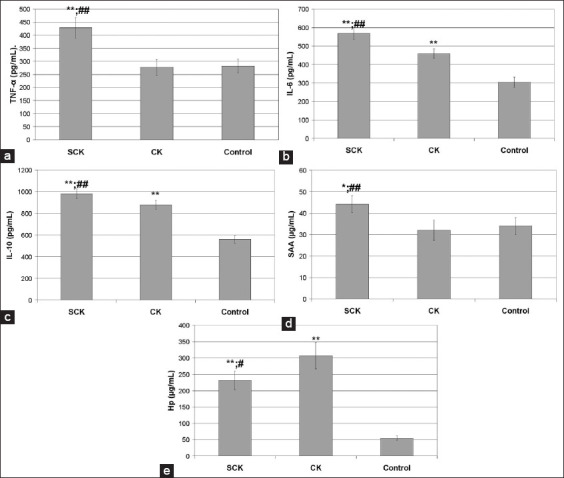
Levels of tumor necrosis factor-α (a), interleukin 6 (IL-6) (b), IL-10 (c), haptoglobin (d), and serum amyloid A (SAA) (e) in blood serum of cows with subclinical ketosis (SCK); clinical ketosis (CK); and control – healthy cows. * – p<0.05; ** – p<0.01 – statistical differences between cows with SCK and CK versus control. # – p<0.05; ## – p<0.01 – statistical differences between cows with SCK versus cows with CK.

BHBA values are presented in Figure-1. In cows with CK, these values were the highest at 4.41±1.19 mmol/L. The values were significantly higher (p<0.001) than those of the group of cows with SCK (1.81±0.24) and the healthy cows (control) (0.9±0.1). Cytokine and APP concentrations in all tested cows are presented in Figure-2. The values of assessed cytokines in SCK cows, such as TNF-α (Figure-2a), IL-6 (Figure-2b), and IL-10 (Figure-2c), and APP, including Hp (Figure-2e), were significantly higher than those of the control group (p<0.01). SAA values (Figure-2d) in this group of cows were also higher than those in the control group, although at a lower level of significance (p<0.05). TNF-α, IL-6, IL-10, and SAA values in the SCK cows were significantly higher than those in the CK cows (p<0.01). Conversely, the SCK group had a lower Hp concentration than had the CK group (p<0.05). The cows with CK had significantly higher values of IL-6, IL-10, and Hp than had the control group (p<0.01).

## Discussion

In this study, the selected parameters of immune system in cows with SCK and CK were assessed. The disease stage was defined on the basis of the quantitative assessment of BHBA, which, according to the literature, is the most useful and reliable ketone body employed for the diagnostics and evaluation of ketosis in dairy cows [6]. Moreover, disease severity was assessed on the basis of the visible clinical signs of ketosis, which is also crucial because it facilitates and accelerates the diagnosis and proper therapeutic procedures. In these study, with respect to the relationship between ketone bodies and immune system, the comparison of concentrations of the assessed parameters between both groups of cows with ketosis seems the most significant because it may be assumed that with the advancement of the disease (BHBA increase), progress is expected in the investigated immune system parameters [22,26]. However, the presented results of this study do not seem to confirm these relationships. Conversely, in our study, the majority of the assessed parameters (TNF-α, IL-6, IL-10, and SAA) were increased in cows with SCK, although not in cows with CK. However, in both groups of cows, the increase of investigated immunological parameters was observed, and this may be a sign of systemic inflammation in these animals. The role of inflammation of the body in ketosis development has not been accurately determined. Whether inflammation may arise earlier and contribute to the development of this disease or whether the increase in inflammation parameters occurs due to the increasing BHBA concentrations in cows remains unknown. Zhang *et al*. [2] showed that inflammation may play a significant role in the development of ketosis in dairy cows. In this study, the cows with ketosis had increased IL-6, TNF-α, and Hp concentrations not only at the moment of diagnosis but also long before, that is, in 8 and 4 weeks before parturition. As shown by the authors, the inflammation develops in the body and is visible in the blood of cows, much earlier before the BHBA increase, in the dry period or very early lactation. Our research seems to confirm and even complement the above results of other authors. We obtained the increased concentration of the investigated immunological parameters in the cows with less severity of the disease (SCK) compared with the cows with more severe ketotic state (CK). If we assume that an untreated SCK can intensify and change into CK, then we can assume that inflammation will occur earlier, before ketosis, contributing to further disease development. It seems to be consistent with the observations of other authors described above. If the increase of immunological parameters was dependent on the concentration of BHBA, their concentration would increase along with the increase of ketone body concentration [2,27]. However, this study does not confirm this relationship. In cows, in which ketosis is deteriorating (BHBA concentration increases and clinical signs appear), the inflammation persists, although at a lower level. Some parameters in cows with CK (TNF-α and SAA) were similar to the parameters in healthy cows (control cows), and other assessed parameters remained at a high level, although significantly lower than those of the SCK cows. On this basis, it can be assumed that the cows with CK had a less pronounced inflammation than had the cows with SCK. Apparently, this is just a presumption, because it has been known from another publication that several other factors can impair an immune cell activity. One of these factors is NEB, which occurs in cows after parturition, wherein ketosis develops as an effect. The accumulation of NEFA and ketone bodies, as an effect of NEB, negatively influences the appetite stimulation in the brain and increases glucocorticoid concentration in the blood, which stimulates the release of other mediators blocking immune functions [28-30]. It seems consistent with our results, in which apart from pro-inflammatory cytokines (TNF-α and IL-6), the anti-inflammatory cytokine (IL-10) was also assessed. The secretion of IL-10 by regulatory lymphocytes has a protective effect and prevents body damage due to the inhibitory effect on immunocompetent effector cells that weaken destructive autoimmune reactions [31]. In this study, the IL-10 concentration was high in both cows with SCK and cows with CK, which shows that in cows with ketosis, inflammatory and anti-inflammatory processes simultaneously run. The significantly increased IL-10 level in SCK cows may contribute to the retardation and inhibition of inflammation in CK cows, as described above. To date, the influence of inflammation, secreted cytokines, and APPs on ketosis and other metabolic diseases development has not been fully understood. Literature data show that IL-6 and TNF-α may influence the development of several diseases, both metabolic and non-metabolic [32-34]. It has been proven that strict correlations exist between the increased IL-6 concentration and lipid and protein metabolism impairment, impaired fatty acid metabolism, and even the occurrence of oxidative stress. Dysfunctions in one or all of the processes mentioned above may lead to ketosis development, liver damage, and failure [32]. According to other authors, IL-6, the increase of which was observed on 33 and 23 days before parturition, may also contribute to the development of other postpartum diseases in dairy cows, such as displaced abomasum or placental retention [33]. Similarly, in humans, TNF-α, as a pro-inflammatory cytokine, is engaged in the pathogenesis of several metabolic diseases, mainly chronic, such as diabetes, contributing to insulin resistance and disturbed fatty acid metabolism [34]. In cows with fatty liver syndrome, the correlation has been described between the TNF-α increase and insulin resistance and disturbed fatty acids metabolism [27,35]. Moreover, IL-6 and TNF may stimulate the breakdown of adipose tissue in an organism through the decrease in fodder consumption, induction of insulin resistance, and direct initiation of lipolysis [36]. These processes are strictly connected with ketosis development in dairy cows [37]. Zhang *et al*. [2] demonstrated not only an IL-6 and TNF-α increase but also an increase in APP concentration in cows 4 weeks before parturition. This suggests the presence of an acute-phase response even before parturition, which is before ketosis development. Serum Hp and SAA are mainly produced by the liver hepatocytes as a response to pro-inflammatory cytokines (e.g., IL-6 and TNF) and glucocorticoids [20,21]. Similar relationships have been observed in the presented study. The consequence of the IL-6 and TNF-α increase in cows with ketosis was the rise in APP concentration. However, in cows with SCK, the increased concentration of both SAA and Hp was obtained, whereas in cows with CK, only the Hp increase was observed. SAA is an apolipoprotein, which appears in the bloodstream as the first response protein up to 24-48 h after the occurrence of an inflammatory factor, such as infection, and its secretion is dependent on IL-1 and/or TNF-α [17,19]. Hp, by contrast, is a protein constituting the second line of response, its secretion is regulated by IL-6, and its high concentration is characteristic of prolonged and mild inflammations [17,19]. This may be an explanation for our results, which show that in cows with SCK, inflammation occurs first, which is why the increased SAA concentrations are noted; however, this condition may last for a different time period before it is diagnosed, and, for that reason, Hp concentrations also increase. In the CK cows, the inflammation lasted longer; therefore, the increased SAA concentrations were not noted, although merely the Hp level was increased compared with that in the SCK cows, which proves the inflammation to be chronic. In the literatures quoted above, the authors did not explain as to why the increase of cytokine and APP levels were observed before parturition in cows [2]. Similarly, in the present study, it is not certain why the cows with SCK had higher listed immunological parameters than the cows with CK. Abuajamieh *et al*. [22] revealed that lipopolysaccharide concentration increases already in the prenatal period, which boosts the activity of immune system (pro-inflammatory cytokines and APPs increase). Lipopolysaccharide may have an origin in the uterus during metritis development, in the udder during mastitis, or in the intestines during leaky gut syndrome. As suggested by some authors, the increased intestinal permeability may be a main cause of inflammation and closely connected to ketosis development in dairy cows. Impaired intestinal integrity may be caused by inappropriate fodder – too much easily fermenting carbohydrates or non-optimal fodder consumption – restriction of the amount of feed given to cows in the drying period [22,38]. On the basis of the results of our own study, it is not possible to unequivocally assess whether inflammation and inflammatory mediators released during ketosis duration may contribute to further ketosis development in dairy cows. However, the comparison of the results of the study by Zhang *et al*. [2], including the high concentration of cytokines and APP even before delivery, with our results, including the higher concentration of cytokines and SAA in cows with SCK than that of cows with CK, suggests that ketosis may develop as a result of inflammation in the cows’ organism e.g., in the udder or other organs, under the influence of secreted inflammatory mediators. This is a novel approach to the disease entity because the majority of authors consider the secreted cytokines and APP as parameters that may be used in the diagnosis of ketosis [26]. However, according to our knowledge, these biomarkers are unspecific and their concentration also increases in several other diseases [16,20,21,31,39]. Only a much earlier prenatal screening can have a diagnostic value regarding ketosis development; however, the confirmation of this screening requires further studies concerning only this issue.

## Conclusion

On the basis of this study, it cannot be unequivocally stated that the ketosis development in dairy cows was preceded by systemic inflammation, and whether the inflammation may affect the ketosis development remains unknown. The concentration of the assessed immunological parameters was not related with the increase in BHBA concentration in the blood serum of cows. This study revealed the increased pro-inflammatory cytokine concentrations, including TNF-α, IL-6, and SAA, and the decreased Hp concentration in the blood serum of cows with SCK compared with that of the cows with CK. This may suggest that the severity of inflammation is greater in a less advanced disease state and decreases as the disease progresses, probably under the influence of anti-inflammatory mechanisms (the increase of IL-10), which protects the organism of an animal from destruction.

## Authors’ Contributions

PB and JM: Obtained funding sources and supervised all stages of the study. PB and JM: Designed the study and participated in patient management, data collection interpretation, and writing of the manuscript. MS and AB: Participated in data collection interpretation and critical revision of the manuscript. UL and HG: Did statistical analysis, data interpretation, preparation, and critical revision of the manuscript. UL and KK: Data collection, interpretation, and writing of the manuscript. All authors have read and approved the manuscript.
